# Venographic Assistance in Peripherally Inserted Central Catheter Placement in a Pediatric Patient: A Case Report

**DOI:** 10.7759/cureus.90697

**Published:** 2025-08-21

**Authors:** Yusuke Tokuda, Hiro Nakao, Shintaro Morooka, Mitsuru Kubota

**Affiliations:** 1 Department of Critical Care Medicine, National Center for Child Health and Development, Tokyo, JPN; 2 Department of General Pediatrics and Interdisciplinary Medicine, National Center for Child Health and Development, Tokyo, JPN

**Keywords:** pediatrics, peripherally inserted central catheter, vascular stenosis, venography, venous thrombosis

## Abstract

Peripherally inserted central catheters (PICCs) are commonly used in pediatric patients for long-term IV access because of their ease of insertion and lower risk of complications such as pneumothorax or hemothorax. However, difficulties may arise when advancing the catheter due to venous stenosis or occlusion. We reported a pediatric case requiring prolonged antibiotic therapy for sacral osteomyelitis, in which PICC placement was initially unsuccessful because of resistance encountered during catheter advancement. Venography revealed significant venous stenosis, which allowed successful catheter placement after the guidewire was redirected into a patent vessel. This case highlighted the utility of venographic assistance in instances of PICC insertion failure.

## Introduction

Peripherally inserted central catheters (PICCs) are increasingly used in pediatric patients to administer parenteral nutrition, high-osmolarity medications, or long-term antibiotics [1]. PICCs, inserted through peripheral veins of the upper extremities, reduce the risk of complications such as pneumothorax and hemothorax [2,3]. However, procedural complications, including arterial puncture, nerve injury, guidewire or catheter advancement failure, and malposition, are not uncommon [2]. Reported success rates for PICC insertion in pediatric populations range from 79% to 96%, with the most frequent reasons for failure being difficulty with vessel access or incomplete guidewire advancement [4-6]. When advancement failed, clinicians were often required to change insertion sites, place a shorter midline catheter, or abandon the procedure. We reported a pediatric case in which venous stenosis impeded catheter advancement, but venographic visualization enabled redirection of the guidewire and successful PICC placement.

## Case presentation

A 10-year-old boy with a history of Chiari malformation type II and lower limb paralysis due to myelomeningocele, previously treated with ventriculopleural shunting, presented with a sacral pressure ulcer exposing bone. He had a history of recurrent infections that required frequent hospitalizations and multiple PICC placements (four in the right arm and two in the left arm). Upon admission for a new episode of fever and sacral ulcer exacerbation, he was diagnosed with sacral osteomyelitis and was deemed to require prolonged IV antibiotic therapy. Therefore, placement of a PICC was planned. A 3 Fr single-lumen PowerPICC^®^ (BD, Franklin Lakes, NJ, USA) was selected, as it was the smallest available size and was anticipated to allow for possible future contrast-enhanced CT. The over-the-wire type was chosen because it provided a rigid and stable conduit for precise catheter insertion over a pre-positioned guidewire.

**Figure 1:**
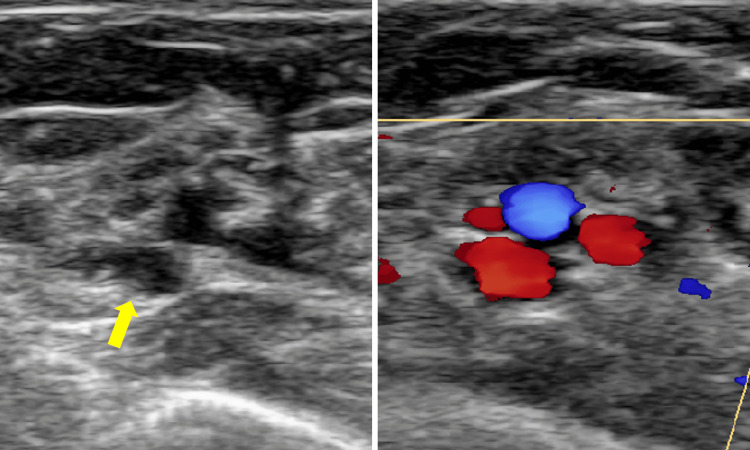
Ultrasound-guided venous evaluation The patency and course of the brachial vein were confirmed by ultrasonography, and the medial brachial vein was selected as the puncture site. Color Doppler demonstrates the veins in red and the adjacent artery in blue.

Before PICC insertion, the upper arm veins were assessed with ultrasound in the fluoroscopy room. The basilic vein was not visualized. The medial brachial vein could not be fully traced proximally on ultrasound; however, color Doppler confirmed blood flow, suggesting it was not completely occluded. As options for peripheral access were minimal, and the brachial vein had an adequate diameter and was located at an accessible depth for puncture, it was selected based on a comprehensive evaluation. The medial brachial vein measured 3 mm in diameter, corresponding to a catheter-to-vein ratio of approximately 33%. Ultrasound evaluation of the veins in the shoulder and neck regions was not performed. Venous access was successfully obtained on the first puncture attempt under real-time ultrasound guidance (Figure 1).

**Figure 2:**
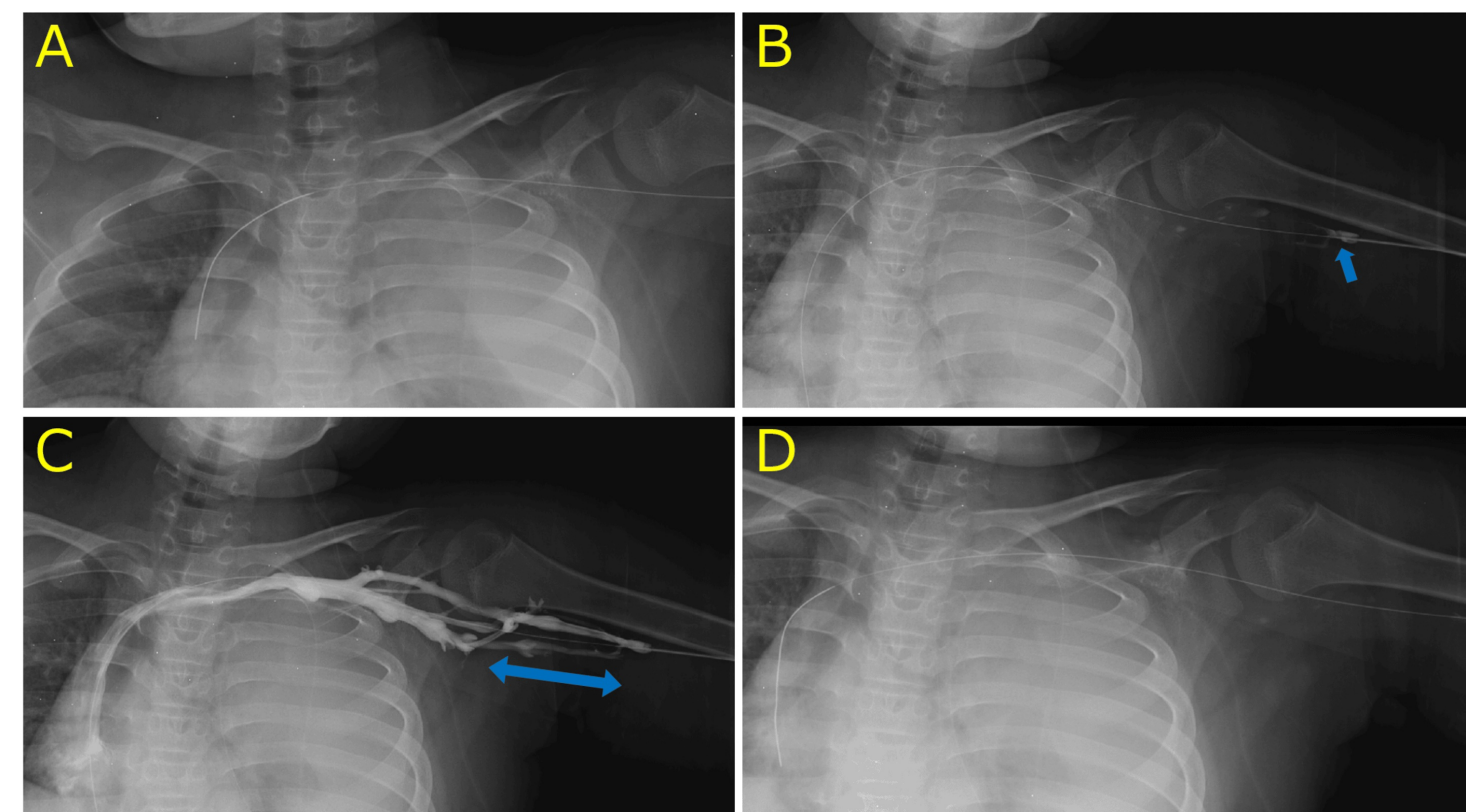
Fluoroscopic images during the procedure Fluoroscopic guidance confirmed the trajectory of the guidewire and catheter. Because of ventriculopleural drainage, there was abundant left pleural effusion, and the left lung was partially collapsed. (A) The guidewire was advanced into the superior vena cava, but the PICC catheter could not pass beyond the proximal upper arm due to resistance. (B) A 3 Fr angiographic sheath was inserted over the guidewire; the arrow indicates the sheath tip. (C) Venography revealed stenosis extending from the medial brachial vein to the axillary vein and opacification of the cephalic vein via a collateral vein on the lateral side. (D) The guidewire was redirected into the cephalic vein and advanced to the superior vena cava.

The guidewire and peel-away sheath introducer advanced smoothly under fluoroscopic guidance using the modified Seldinger technique; however, the PICC catheter could not pass beyond the proximal upper arm due to resistance (Figure 2A). After removal of the peel-away sheath introducer and PICC catheter, a 3 Fr angiographic sheath was introduced over the guidewire (Figure 2B). Venography revealed stenosis extending from the medial brachial vein to the axillary vein. This segment was considered almost completely stenotic, as contrast was not visualized within it, although injection demonstrated opacification of the cephalic vein via a collateral vein (Figure 2C). The angiographic sheath was partially withdrawn just proximal to the collateral vein bifurcation, and the guidewire was redirected into the cephalic vein and advanced to the superior vena cava (Figure 2D). The PICC catheter was then reinserted without resistance and positioned successfully. The procedure was completed without changing the puncture site, and no contrast-related allergic reactions or renal dysfunction were observed post-procedure. The PICC remained functional for more than one month, during which no catheter-related bloodstream infection or newly developed thrombosis was noted.

## Discussion

In this case, venous stenosis obstructed catheter advancement, and venographic guidance enabled successful guidewire redirection and catheter placement. Venography provides direct visualization of venous anatomy and pathology, such as stenosis, and facilitates procedural planning and execution [7]. It also allows identification of venous anomalies and collateral circulation, enabling the planning of alternate access routes during the procedure. When preprocedural assessment of vascular patency is insufficient, blind attempts at guidewire redirection may fail. In our case, venography likely reduced procedural time and radiation exposure by promptly identifying a viable venous pathway.

The venous stenosis in this patient was likely the result of prior PICC-related thrombosis. Previous studies have identified multiple PICC insertions, high catheter-to-vein diameter ratios, and prolonged dwell times as risk factors for thrombosis in pediatric patients [3,8]. Moreover, the likelihood of requiring venographic assistance or a change in insertion site increases with the number of prior PICCs [9]. Although ultrasound is the gold standard for assessing catheter-related thrombosis [10], in pediatric patients, smaller vessel diameters and limited visibility make it difficult to evaluate patency up to the central veins, especially when stenosis or occlusion is present. For patients with multiple prior PICC placements, careful evaluation of venous patency is therefore essential, and venography should be considered when obstruction obscures the venous pathway.

The risks of venography include contrast-induced allergic reactions and nephrotoxicity [11], as well as radiation exposure and increased procedural costs. Thus, venography should be regarded as a supplemental tool. Alternatives include using smaller-diameter catheters or selecting a different insertion site; however, each carries inherent limitations. Smaller-caliber catheters may not support high infusion rates, and when placed in stenotic veins, even these may cause venous stasis, thrombosis, or difficult removal due to reduced clearance between the catheter and the vessel wall. Changing the insertion site requires another venipuncture, which increases the risk of hematoma or phlebitis and may compromise future access [12]. In patients with limited venous access, as in this case, preservation of viable veins is crucial. Successful completion of the procedure without changing the puncture site may help preserve long-term vascular access.

## Conclusions

This case demonstrates that venographic assistance can facilitate PICC placement in pediatric patients with suspected venous stenosis, particularly those with multiple prior PICC insertions or limited peripheral venous access. Ultrasound evaluation should be performed first, and venographic assistance should be considered when significant obstruction or stenosis is identified and catheter advancement is expected to be difficult.
